# Sonication, Vacuum Infiltration and Thiol Compounds Enhance the *Agrobacterium*-Mediated Transformation Frequency of *Withania somnifera* (L.) Dunal

**DOI:** 10.1371/journal.pone.0124693

**Published:** 2015-04-30

**Authors:** Ganeshan Sivanandhan, Gnajothi Kapil Dev, Jeevaraj Theboral, Natesan Selvaraj, Andy Ganapathi, Markandan Manickavasagam

**Affiliations:** 1 Plant Molecular Biology Laboratory, Department of Biotechnology and Genetic Engineering, School of Biotechnology, Bharathidasan University, Tiruchirappalli 620 024, Tamil Nadu, India; 2 Plant Tissue Culture Laboratory, Department of Botany, Periyar E.V.R College (Autonomous), Tiruchirappalli 620 023, Tamil Nadu, India; 3 Molecular Genetics and Genomics Laboratory, Department of Horticulture, College of Agriculture and Life Sciences, Chungnam National University, Daejeon 305 764, South Korea; University of Minnesota, UNITED STATES

## Abstract

In the present study, we have established a stable transformation protocol via *Agrobacterium tumafacines* for the pharmaceutically important *Withania somnifera*. Six day-old nodal explants were used for 3 day co-cultivation with *Agrobacterium tumefaciens* strain LBA4404 harbouring the vector pCAMIBA2301. Among the different injury treatments, sonication, vacuum infiltration and their combination treatments tested, a vacuum infiltration for 10 min followed by sonication for 10 sec with *A*. *tumefaciens* led to a higher transient GUS expression (84% explants expressing GUS at regenerating sites). In order to improve gene integration, thiol compounds were added to co-cultivation medium. A combined treatment of L-Cys at 100 mg/l, STS at 125 mg/l, DTT at 75 mg/l resulted in a higher GUS expression (90%) in the nodal explants. After 3 days of co-cultivation, the explants were subjected to three selection cycles with increasing concentrations of kanamycin [100 to 115 mg/l]. The integration and expression of *gusA* gene in T0 and T1 transgenic plants were confirmed by polymerase chain reaction (PCR), and Southern blott analysis. These transformed plants (T0 and T1) were fertile and morphologically normal. From the present investigation, we have achieved a higher transformation efficiency of (10%). Withanolides (withanolide A, withanolide B, withanone and withaferin A) contents of transformed plants (T0 and T1) were marginally higher than control plants.

## Introduction

*Withania somnifera* (L.) Dunal (Solanaceae) has gained a significant attention because of its therapeutic phytocompounds “withanolides” [1, 2, 3]. *In vitro* and *in vivo* pharmacological investigations elucidated that the curative properties of this plant have been associated with withanolides which are found in leaves and roots [4]. The concentration of withanolides localized in leaves usually ranges from 0.001 to 0.5% of dry weight [5]. Although, conventional breeding helped *W*. *somnifera* improvement and phytochemical production, it has its own limitations such as a long breeding cycle, narrow genetic base, susceptibility to environmental factors and so on [6]. The sum of all these factors causes difficulties in the compositional standardization of herbal formulation and the commercial exploitation of this plant [7]. In spite of all these problems, there has been an ever-increasing demand for *W*. *somnifera* in larger quantities every year [3].

Metabolic engineering of secondary metabolites is envisaged as an effective and powerful tool for improving the biosynthesis of therapeutically useful compounds in medicinal plants [8, 9]. Previously, we have undertaken different attempts in order to improve the withanolides content in cell or organ cultures of *W*. *somnifera* [2, 3, 10, 11, 12–14]. Yet, the most economical system to yield the drug on a large scale appears to be by means of transgenic plants [15]. Information concerning *Agrobacterium*-mediated genetic engineering of *W*. *somnifera* has been scanty. Ray and Jha [16] obtained only shooty and rooty teratomas by using wild type strain of *Agrobacterium tumefaciens* whereas Pandey et al. [17] achieved a transformation efficiency of 1.67% using LBA4404 containing the binary vector pIC121Hm. Recently, progress in the molecular regulation of withanolides biosynthesis has been elucidated [18, 19]. The genes of the key enzymes involved in the biosynthesis of withanolides, such as *farnesyl pyrophosphate synthase* (FPPS), *squalene synthase* (SQS), *squalene epoxidase* (SQE), *1-deoxy-D-xylulose-5-phosphate synthase*, *1-deoxy-D-xylulose-5-phosphate reductase* and *sterol methyltransferase* (SMT) have been cloned from *W*. *somnifera* [18, 19]. The method developed in the present study may be useful to over express the enzymes involved in withanolides biosynthesis pathway in the transgenic plants of *W*. *somnifera*. The use of sonication and vacuum infiltration has not been reported in earlier *W*. *somnifera* transformation studies [16, 17]. The addition of thiol compounds such as L-cysteine (L-Cys), sodium thiosulphate (STS) and dithiothreitol (DTT) to co-cultivation medium upon *Agro*-infection reduced wound- and increased pathogen defense response and the potentiality of *Agrobacterium* to infect plant tissue [20]. Hence, a comprehensive approach has been made in the present investigation to standardize various paramaters such as OD, virulence inducer, infection strategy, co-cultivation period, bactericidal antibiotics, sonication time, vacuum infiltration time, and addition of thiol compounds for developing optimal transformation system for *W*. *somnifera*.

## Materials and Methods

### Ethics statement

The complete genetic transformation study has been submitted to our University Institutional Biosafety Committee and duly approved by the committee members. The committee recommendations has been sent to Department of Biotechnology, Govt. of India and approved by authorities (Approval No.: BT/BS/17/29/2000-PID dated 14.05.2009). All the putative transgenic plants-raised from in vitro conditions have been transferred to controlled Green house of Department of Biotechnology and Genetic Engineering, Bharathidasan University. The Green house and plant maintenance were completed in accordance with the rules and regulations recommended for transgenic experiments by Department of Biotechnology, Govt. of India. We would like to ascertain that no plants/propagules were transferred to open field conditions. The complete transgenic experiments were conducted in Bharathidasan University owned by Tamil Nadu State Government, India. No animals or pathogens were involved in the present research.

### Plant material and regeneration

Three-month-old nodal explants of field-grown *W*. *somnifera* were collected and surface sterilized by following the method of Sivanandhan et al. [10]. The nodal explants (10 mm) were inoculated onto MS [21] solid medium containing 1.5 mg/l BA, 0.3 mg/l IAA and 20 mg/l spermidine for multiple shoot induction (WsSIM) [Table 1]. This hormonal variant was selected on the basis of our earlier report [10]. The cultures were incubated at 25±2°C under a 16-h photopheriod (50 μmol m^–2^ s^–l^).

**Table 1:** Media used in the present investigation.

Media	Uses	Composition
WsSIM	Shoot induction	MS medium, 1.5 mg/l BA, 0.3 mg/l IAA, 20 mg/l spermidine, 0.2% phytagel, 3% sucrose, pH 5.75
LWsSIM	Liquid shoot induction medium	MS medium, 1.5 mg/l BA, 0.3 mg/l IAA, 20 mg/l spermidine, 3% sucrose, pH 5.75
WsRIM	Root induction	MS medium, 2 mg/l IBA, 20 mg/l putrescine, 0.2% phytagel, 2% sucrose, pH 5.75
WsSIM+AS	Co-cultivation medium	WsSIM, AS 150 μM, 0.6% phytagel, 3% sucrose, pH 5.8, 20 mM MES
WsSIM+L-Cys+AS	Co-cultivation medium	WsSIM, L-cysteine (100 mg/l), AS 150 μM, 0.6% phytagel, 3% sucrose, 20 mM MES, pH 5.8
WsSIM+DTT+AS	Co-cultivation medium	WsSIM, Dithiothreitol (75 mg/l), AS 150 μM, 0.6% phytagel, 3% sucrose, 20 mM MES, pH 5.8
WsSIM+STS+AS	Co-cultivation medium	WsSIM, sodium thiosulphate (125 mg/l), AS 150 μM, 0.6% phytagel, 3% sucrose, 20 mM MES, pH 5.8
WsSIM+L-Cys+DTT+STS+AS	Co-cultivation medium	WsSIM, L-Cys (100 mg/l), DTT (125 mg/l), STS (75 mg/l), AS 150 μM, 0.6% phytagel, 3% sucrose, 20 mM MES, pH 5.8,
LWsWM	Liquid washing medium	WsSIM, timentin (150 mg/l), pH 5.75
WsSSM-I	Shoot selection medium-I	WsSIM, kanamycin (100 mg/l), timentin 150 mg/l, pH 5.75
WsSSM-II	Shoot selection medium-II	WsSIM, kanamycin (110 mg/l), timentin 150 mg/l, pH 5.75
WsSSM-III	Shoot selection medium-III	WsSIM, kanamycin (115 mg/l), timentin 150 mg/l, pH 5.75
WsRSM	Root selection medium	WsRIM kanamycin (75 mg/l), timentin 150 mg/l, pH 5.75

### Effect of cefotaxime, timentin and augmentin on nodal regeneration

Prior to *Agrobacterium*-mediated transformation experiment, to determine the exact effect of antibiotics’s type and concentration on shoot regeneration, the nodal explants from *in vitro*-regenerated shoots were placed on shoot induction medium (WsSIM) containing different concentrations of filter-sterilized cefotaxime, timentin or augmentin 0–450 mg/l ranging from 0, 100, 150, 200, 250, 300, 350, 400 and 450 mg/l in a Petri dish (100×25 mm; 30 ml medium). The shoot regeneration response was evaluated under the phytotoxic conditions after 4 weeks of culture *in vitro*. All cultures were incubated at 25±2°C under a 16-h photopheriod (50 μmol m^–2^ s^–l^).

### Sensitivity of nodal explants to kanamycin

An optimal concentration of kanamycin for the selection of transformed shoots was determined by culturing non-transformed nodal explants on WsSIM fortified with different concentrations of kanamycin (0–250 mg/l) and necrosis of the plant tissues was documented after 4 weeks. The cultures were transferred to the same fresh medium containing the same level of antibiotics after every two weeks still a total of 4 weeks and then estimated for shoot regeneration responses. Similarly, in an another experiment, a kanamycin (0–125 mg/l) concentration that suppressed root induction was evaluated by transferring the elongated shoots (appx. 4–5 cm in length) regenerated from non-transformed explants on WsRIM. The cultures were incubated at 25±2°C under a 16-h photopheriod (50 μmol m^–2^ s^–l^).

### *Agrobacterium* strain and plasmid vector

**Fig 1:**
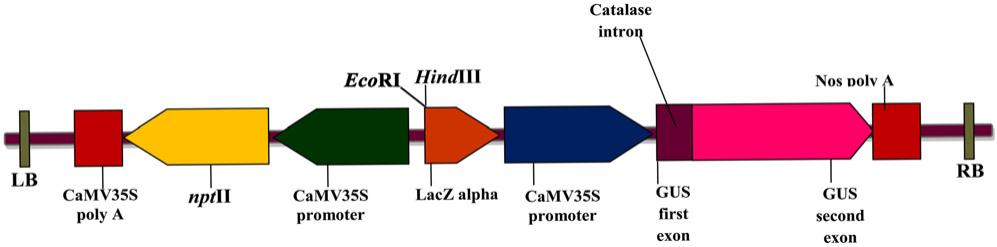
Schematic representation of genes between T-DNA right border (RB) and left border (LB) of the gene construct pCAMBIA 2301 used for Agrobacterium-mediated transformation of W. somnifera. The nptII gene is presented for kanamycin resistance under the control of the CaMV 35S promoter (left) and the gusA gene is used as a scorable marker under the control of the CaMV35S promoter (left).

*A*. *tumefaciens* strain LBA4404 was used for the transformation studies, harboring the binary vector pCAMBIA 2301, which carries the *npt*II gene and *β-glucuronidase* (*gusA*), reporter genes with a catalase intron between the first and second exons (CAMBIA, Canberra, Australia). Both genes are under control of the CaMV 35S promoter and poly (A) terminator (Fig 1). The binary vector was mobilized into *Agrobacterium* strain by tri-parental mating using pRK2013 as helper vector [22]. *A*. *tumefaciens* strain was maintained on petri plates containing AB agar medium supplemented with 10 mg/l rifampicin (Sigma, St Louis, USA) and 50 mg/l kanamycin. A single colony of *Agrobacterium* was suspended in 5 ml yeast extract and peptone medium (YEP) containing 50 mg/l kanamycin and 10 mg/l rifampicin. After 24 h, 500 μl of this culture was transferred to 25 ml AB minimal medium, pH 7.0, containing 50 mg/l kanamycin and 10 mg/l rifampicin and incubated at 200 rpm on a rotary shaker at 28°C. The culture was pelleted at 8,000 rpm, 28°C and the resulting pellet was re-suspended in 25 ml LWsSIM (pH 5.7) with 150 μM acetosyringone and 20 mM MES.

### *Withania* transformation

Prior to *Agrobacterium* infection, to optimize the pre-culture duration of the explants, the nodal explants (0.5 cm) derived from regenerated shoots (regenerated from field-grown nodal explants) were precultured on WsSIM medium for different time durations (0, 4, 6, 8 and 10 days). After preculture durations, the mersitematic region of nodal explants was injured slightly by pricking 1–2 times with a sterile hypodermic needle (27G1/2) and the injured nodal explants were transferred to petri plates containing 25 ml of bacterial inoculum with different OD_600_ of 0.1, 0.2, 0.5 and 1.0 to access the *Agrobacterium* culture density on *Withania* transformation, placing the meristematic region of the nodal explants down into the inoculum. The plates were gently rotated for 5 min in bacterial inoculum. After immersion of the explants in the different bacterial inoculum, the infected explants were blot dried using sterile Whatman No. 1 filter paper for 10 min and then the nodal explants were transferred to co-cultivation medium. The infected explants on WsSIM were subjected to different co-cultivation days (0, 1, 2, 3, 4 and 5 days). After optimization of co-cultivation days, the injured nodal explants were co-cultivated on WsSIM+AS containing various concentrations of acetosyringone (AS; 0–175 μM). In order to increase the transient GUS expression frequency, the injured-nodal explants were subjected to a different sonication time regime (0–25 Sec) separately in addition with hand pricking (1–2 times) in LWsSIM at a constant frequency of 30 kHz. After 7 days (resting period), the sonicated explants were transferred to WsSSM-I containing 100 mg/l kanamycin for 15 days. After the standardization of sonication treatment, nodal explants with hand pricking were subjected to various periods (0–20 min) of vacuum infiltration (at 75 Hg) separately in LWsSIM to improve the transient GUS expression frequency in *W*. *somnifera*. After optimization of sonication and vacuum infiltration, the optimal ranges of the respective treatments were combined and examined their effect on transient GUS expression frequency on the same medium. All the nodal explants subjected to different physical treatments were co-cultivated for 3 days on WsSIM+AS containing 150 μM acetosyringone. Still the transformation efficiency was lower upon application of AS addition to co-cultivation medium, sonication and vacuum infiltration treatment to nodal explants. Four types of co-cultivation media containing thiol compounds (WsSIM+L-Cys+AS, WsSIM+DTT+AS, WsSIM+STS+AS, and WsSIM+L-Cys+DTT+STS+AS; see Table 1) were investigated to increase the transformation experiment. Control cultures were maintained throughout the experiment. After various physical and chemical treatments on co-cultivation medium in all the experiments, the nodal explants were first washed with sterile distilled water for 3 times, and then with LWsWM containing 150 mg/l timentin. Then the nodal explants were dried and inoculated on WsSIM supplemented with 150 mg/l timentin for 7 days resting period. After that, the explants were subjected to three selection cycles to recover the transgenic shoots on WsSSM. First cycle was carried out on WsSSM-I supplemented with 100 mg/l kanamycin for 15 days. After the first cycle of 15 days, the nodal explants resisted on WsSSM-I were again subjected to a second cycle on WsSSM-II containing 110 mg/l kanamycin for another 15 days. Subsequently, third cycle was passed out on WsSSM-III containing 115 mg/l kanamycin for another 15 days. After 45 days with passing off three rounds of selection cycle, a group of shoots that attained an average length of 4–5 cm on WsSSM-III, they were cut off for root induction on WsRSM with kanamycin for 4 weeks. The transformed putative elongated-shoots were transferred to MS basal medium without any selection agent for 10 days. After the time duration, the selection agent was gradually increased from 50 mg/l to 75 mg/l kanamycin to effectively induce and screen the transformed roots from the elongated shoots with 10 days interval. Subsequently, first selection cycle in WsSSM-I, the nodal explants were tested for transient GUS expression. The stable GUS expression frequency was recorded for multiple shoots after IIIrd selection cycle. Next to root induction from transformed shoots, they were analysed for GUS expression after 4 weeks. The stable GUS expression frequency was recorded for multiple shoots which were grown in optimized antioxidant supplemented medium after III^rd^ selection cycle; after root induction from transformed shoots, they were analyzed for GUS expression after 4 weeks.

### Establishment of transgenic plants

After 4 weeks, well rooted transgenic plantlets were removed from the WsRSM and the roots were washed gently under running tap water to remove the adhering solidifying agent. The plantlets were then transferred to paper cups (7.5 cm diameter) containing autoclaved vermiculite, garden sand and soil mixture (1:1:2 v/v/v) moistened initially with ¼ inorganic salts of MS medium an every alternate days for 3 weeks followed by tap water. The plantelets were covered with a transparent polyethylene bag with minute puncher and maintained inside the plant growth chamber set at 25±2°C, 85–95% relative humidity (RH) and a 16-h photoperiod with light provided by cool-white fluorescent tubes at an intensity of 50 μmol m^–2^ s^–l^. After 3 weeks, all the plants (T_0_) were transferred to large clay pots (20 cm diameter) containing the same substrate mixture and transplanted to the greenhouse.

### Histochemical assay for β-glucuronidase

GUS assay was carried out to confirm the presence of the introduced genes in the transformed tissues according to Jefferson et al. [23]. Tissue was incubated in a staining solution (0.1 M NaHPO_4_ buffer (pH 7.0), 0.5 mM K_3_[Fe(CN)6], 0.5 mM K_4_[Fe(CN)6], 10 mM EDTA, 800 mg/l X-Gluc (5-bromo-4-chloro-3-indolyl β-D-glucuronide), 0.06% (v/v) Triton X-100). The reaction was carried out overnight at 37°C. The chlorophyll was removed from the plant tissues using 99.9% (v/v) ethanol for 6 h prior to visualization and photography.

### Molecular confirmation

#### Polymerase Chain Reaction (PCR) analysis

DNA from T_0_ plants that survived on kanamycin selection, T_1_ plants and non-transformed plants was isolated by the CTAB method [24]. PCR analysis was performed with the genomic DNA to check for the presence of transgene in the transformants using primers for *gusA* gene. The *gusA* gene fragment (515 bp) was amplified (primers 5-ATTGATCAGCGTTGGTGG-3’ and 5’-ACGCGTGGTTACAGTCTTGC-3’) using a thermal cycler (MJ Research, Waltham, MA, USA) with the program: 30 cycles (first heated at 94°C for 5 min, 94°C for 1 min, 55°C for 1 min, 72°C for 1 min) and by a 7-min final extension at 72°C. The master mix for the PCR contained 10 pmol of each primer, 10 mM of each dNTP and 2 U Taq DNA polymerase, 15 mM MgCl_2_, and 50 ng DNA. Plasmid DNA from pCAMBIA 2301 and non-transformed plants genomic DNA were used as a positive and negative controls, respectively. The amplified products were analyzed by electrophoresis on a 1% (w/v) agarose (SRL Pvt. Ltd) gel.

#### Southern blot hybridization

Genomic DNA samples (10 μg) from PCR-positive T_0_ and T_1_ plants, non-transformed plants, and the binary vector pCAMBIA 2301 (5.0 μg) were digested overnight with *Eco*RI, and the resulting fragments were separated by electrophoresis on a 1% (w/v) agarose gel. The size fractionated DNA fragments were transferred to a Hybond N^+^ membrane (GE healthcare, Buckinghamshire, UK) according the method described by Sambrook et al. [25]. The blot was hybridized with an alkaline phosphatase (ALP)-labelled, PCR amplified *gusA* fragment (515 bp) at 55°C for 8 h. The hybridized membrane was washed according to manufacturer’s instructions (GE healthcare), subjected to chemiluminescent development using CDP-Star substrate, and exposed to X-ray film (Kodak, Mumbai, India). The binary vector pCAMBIA 2301 plasmid and genomic DNA from wild type plantlets served as positive and negative controls, respectively.

### Withanolides extraction and HPLC analysis

Transgenic (T_0_ and T_1_) and non-transgenic plants were dried and ground into fine powder (1 g DW). Extraction and HPLC analysis were performed as described by Sivanandhan et al. [12]. The relative amounts of withanolides were calculated by comparing their peak areas with standard curve generated using different amounts of external standards. Withanolides data were expressed as milligram per gram dry weight. Each sample was run in triplicate manner to check the consistency over the previous results. Standard samples of withanolide A and B were obtained from Chromadex Inc. (Laguna Hills, CA, USA) and withaferin A and withanone were obtained from Natural Remedies (Bangalore, Karnataka, India).

### Statistical analysis

The data were analyzed using one-way ANOVA. The mean values of the treatments were subjected to Duncan’s multiple range test (DMRT). The significance was determined at p<0.05 using SPSS 17.09 software for Windows 8.

## Results and Discussion

### Plant material and regeneration

In our previous study, we established that nodal explant was an ideal tissue for the production of higher number of multiple shoots (46 shoots/explant) of *W*. *somnifera* [10] and hence, this protocol was adopted for the production transgenic plants.

### Sensitivity of nodal explants to cefotaxime, timentin and augmentin

**Fig 2:**
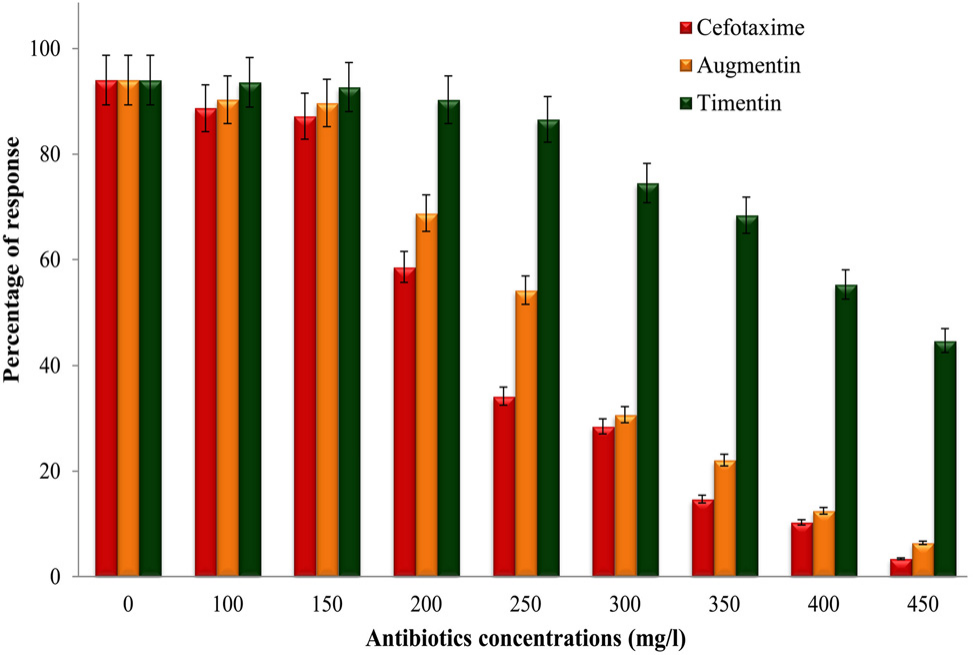
Effect of various antibiotics concentrations on the regeneration ability of nodal explants of *W*. *somnifera*. Data were obtained after 4 weeks for the percentage of response of nodal explants. Each value represents the mean of three independent experiments with 25 explants per treatment.

Fig 2 illustrates the optimum concentration of cefotaxime, timentin and augmentin to control *Agrobacterium* over growth without inhibiting shoot regeneration after 4 weeks of culture. The response of nodal explants for shoot induction on WsSIM containing either cefotaxime or timentin or augmentin was significantly lower than that of nodal explants cultured on an antibiotics-free WsSIM (control). The nodal explants showed no obvious morphogeneic difference on WsSIM without cefotaxime or timentin or augmentin. At a concentration of 300 mg/l cefotaxime, 200 mg/l timentin or 350 mg/l augmentin, the nodal explants showed 28%, 90% and 22% shoot regeneration response respectively, and at a concentration of 400 mg/l cefotaxime, 300 mg/l timentin or 450 mg/l augmentin, the responses were 10%, 74% and 6% for shoot regeneration respectively (Fig 2). From the present study, timentin at 150 mg/l showed an effective bacterial elimination without adversely affecting the morphogenesis of nodal explants when compared to cefotaxime or augmentin treatment. Hence, timentin was incorporated in the WsSIM to check *Agrobacterium* growth after co-cultivation, and at this concentration, the shoots appeared healthy. Augmentin (400 mg/l) was earlier employed for *Agrobacterium*-mediated transformation studies in *W*. *somnifera* [17] using leaf explants derived from the greenhouse plants. However, our study showed that higher than 150 mg/l augmentin was detrimental to the explants from *in vitro-*raised shoots. Timentin had a minimal effect on plant regeneration compared to cefotaxime, which frequently induced hyperhydration of explants, reducing the plant regeneration frequency [9].

### Sensitivity of nodal explants to kanamycin

It was observed that with an increase in kanamycin concentration, the nodal explants resulted in a significant decline in the survival response and regeneration due to antibiotic toxicity (Table 2). Intense browning of nodal explants due to cell death was observed at the concentration of 100 mg/l kanamycin. The nodal explants cultured in control (without selection agents) exhibited healthy, viable shoots and showed 94% survival. However, in the present investigation, three cycles of selection were employed with increasing selection regimes of 100,110,115 mg/l kanamycin which not only reduced the frequency of escapes but also frequency of transformants. Based on this study, 100 mg/l kanamycin was selected to allow the selective growth of transformed shoots and to kill non-transgenic shoots. Experimental results showed that the presence of kanamycin in the WsRIM caused considerable toxicity in the elongated-shoots and reduced their root induction potential when compared to those cultured on kanamycin free media. In the medium without kanamycin (control), maximum rooting response (100%) was noticed. An increase of kanamycin concentration from 25 to 100 mg/l in WsRIM, resulted in the progressive prevention of root induction response, and complete inhibition of root production was observed at 75 mg/l concentration of (Table 2). Therefore, 75 mg/l kanamycin was selected as sensitive concentration for root induction of elongated shoots.

**Table 2:** Effect of kanamycin on sensitivity of nodal explants to regeneration and elongated shoots of *W*. *somnifera* on root induction.

Kanamycin concentration (mg/l)	#Percentage of regeneration	*Percentage of rooting
0	94.2a	100.0a
25	88.4b	74.5b
50	37.6c	28.4c
75	0.0d	0.0d
100	0.0d	0.0d
125	0.0d	0.0d
150	0.0d	-
200	0.0d	-
250	0.0d	-

Control (0): treatments without antibiotics

#Nodal explants were inoculated on WsSIM with different concentrations of kanamycin. Data were collected after 4 weeks of culture.

#Values represent means for 25 explants per treatment, replicated five times. Means with common letters are not significantly different at *p*≤0.05 according to Duncan’s multiple range test (DMRT).

*Elongated shoots were inoculated on WsRIM with different concentrations of kanamycin. Data were collected after 4 weeks of culture.

*Values represent means for 25 elongated shoots per treatment, replicated five times. Means with common letters are not significantly different at *p*≤0.05 according to Duncan’s multiple range test (DMRT).

### *Withania* transformation

#### Effect of bacterial inoculum on transient GUS Expression

**Fig 3:**
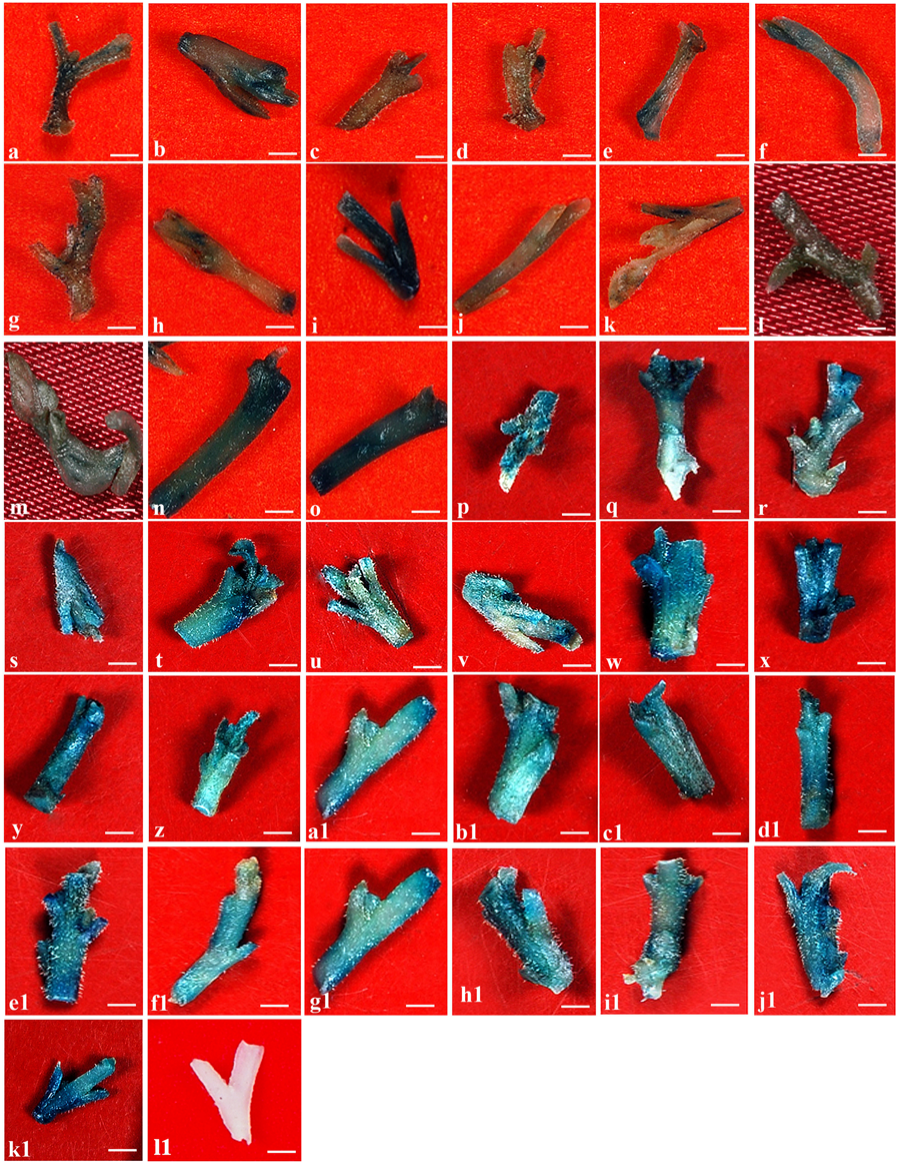
Transient GUS expression in regeneration site of nodal explants of *W*. *somnifera* under different parameters tested. Effect of culture OD_600_ at 0.1 (a), 0.2 (b), 0.5 (c) and 1.0 (d); effect of AS concentrations-e. 50 μM, f. 75 μM, g. 100 μM, h. 125 μM, i. 150 μM, and j. 175 μM; effect of sonication treatment-k. 5 sec, l. 10 sec, m. 15 sec, n. 20 sec, o. 25 sec; effect of vacuum infiltration-p. 5 min, q. 10 min, r. 15 min, s. 20 min; t. combined effect of sonication (10 sec) and vacuum infiltration (10 min); effect of L-cysteine concentrations- u. 100 mg/l, v. 200 mg/l, w. 300 mg/l, x. 400 mg/l, y. 500 mg/l; effect of STS concentrations- z. 50 mg/l, a1. 75 mg/l, b1. 100 mg/l, c1. 125 mg/l, d1. 150 mg/l, e1. 175 mg/l; effect of DTT concentrations—f1. 25 mg/l, g1. 50 mg/l, h1. 75 mg/l, i1. 100 mg/l, j1. 125 mg/l; k1. Combined effect of L-cysteine (100 mg/l), STS (125 mg/l) and DTT (75 mg/l); l1. Control explant. The GUS expression was performed on 6 day pre-cultured nodal explants after 3 days co-cultivation

The bacterial cell density used for *Agro*-infection of explants significantly improved the binding of T-DNA delivery to the cells. Maximum transient GUS expression (64%) was obtained in the nodal explants which were infected with a bacterial density 0.2 (OD_600_) (Table 3; Fig 3a). When the bacterial density was increased to 0.5 or above, the GUS expression frequency was reduced. Higher densities of *Agrobacterium* increased toxins to the receptor cells [26]. In plant transformation, many reports emphasized the optimal cell density of *Agrobacterium* in the range between 0.1–1. From the present study, it was demonstrated that determining the optimal cell density was important because, at high cell densities, the nodal explants were fully colonized by *Agrobacterium* and resulted in explant death (data not shown). Sonia et al. [27] observed that the tissue regeneration ability and GUS expression were decreased due to increased production of toxic compounds owing to bacterial overgrowth, which resulted in the necrosis of explant’s tissue.

**Table 3:** Factors that affect the transient expression of *gusA* gene in nodal explants of *W*. *somnifera* infected with *A*. *tumefaciens* strain LBA4404 containing vector pCAMBIA2301.

Different factors with variables	Transient GUS expression frequency (%)
^a^Pre-culture durations (days)	
0	26.8h
4	47.3g
6	63.4e
8	55.7f
10	40.0g
^a^Bacterial density (OD_600_)	
0.1	56.7f
0.2	64.2e
0.5	47.3g
1.0	28.7h
^a^co-cultivation period (days)	
0	0i
1	45.0g
2	53.5f
3	64.6e
4	43.2g
5	27.5h
^b^AS (μM)	
0	64.6e
50	66.5e
75	68.7e
100	70.3d
125	72.6d
150	74.2d
175	71.0d
^c^Sonication treatment (sec)	
0	74.2d
5	75.6d
10	76.0d
15	77.5d
20	78.6d
25	100.0a
^d^vacuum infiltration (min)	
0	78.6d
5	79.3d
10	81.5c
15	94.8b
20	100.0a
^e^Sonication (10 sec)+vacuum infriltration (10 min)	84.0c
^f^Thiol compounds (mg/l)	
L-Cys	
100	87.6c
200	88.5c
300	90.4b
400	92.2b
500	93.6b
STS	
50	71.1d
75	76.6d
100	79.7d
125	82.6c
150	84.5c
175	86.4c
DTT	
25	69.4e
50	74.3d
75	78.8d
100	80.3c
125	83.8c

Control (0): Treatment without factors

^a^Nodal explants from different time duration were pre-cultured on WsSIM and hand-pricked (1–2 times) nodal explants were infected with different *Agro* culture densities and the explants were co-cultivated for different days on WsSIM medium.

^b^The effect of AS was evaluated by incorporating it in WsSIM+AS medium. Six-day old pre-cultured nodal explants were infected at OD_600_ 0.2 of *Agro* culture and co-cultivated for 3 days on WsSIM+AS containing various concentrations of AS.

^c^Six-day old precultured nodal explants were infected in the presence of sonication at different time durations at OD_600_ 0.2 of *Agro* culture and co-cultivated for 3 days on WsSIM+AS (150 μM).

^d^Six-day old pre-cultured nodal explants were infected in the presence of vacuum infiltration at different time durations at OD_600_ 0.2 of *Agro* culture and co-cultivated for 3 days on WsSIM+AS (150 μM).

^e^Six-day old pre-cultured nodal explants were infected at OD_600_ 0.2 of *Agro* culture with a combination of sonication (10 sec) + vacuum infiltration (10 min) and co-cultivated for 3 days on WsSIM+AS (150 μM).

^f^Six-day old pre-cultured nodal explants were infected and sonicated (10 sec) and vacuum infiltrated (10 min) at OD_600_ 0.2 of *Agro* culture and co-cultivated for 3 days on WsSIM+AS (150 μM) containing various concentrations of thiol compounds.

Data were collected at the end of I^st^ selection cycle on WsSSM-I (15^th^ day). The explants subjected to different factors were placed on WsSIM for 6 days for resting and the explants were transferred to WsSSM-I at 7^th^ day.

### Effect of pre-culture duration on transient GUS expression

The effect of pre-culture duration on transformation efficiency was assessed by subjecting the nodal explants on pre-culture medium (WsSIM). The efficiency of transient GUS expression declined with increasing pre-culture duration. The 6-day-old nodal explants showed a maximum GUS expression of 63% at 0.2 OD of *Agrobacterium* suspension (Table 3; Fig 3b). High transformation efficiency on younger tissues could be due to the fact that many cells in young tissue are in active state of division and hence are susceptible to T-DNA integration to a greater extent compared to older tissues [28]. Bond and Roose [29] reported that the differences in transformation efficiency between younger and older tissues could also be attributed to differences in cell wall composition, which may influence bacterial binding. In earlier reports, pre-culture of the explants on an appropriate medium prior to *Agro* infection has been reported to improve the transformation efficiency in *Sesbania drummondii* [30] and in *Lycopersicum* [31].

### Effect of co-cultivation duration on transient GUS expression

Co-cultivation is one of the crucial factors influencing *Agrobacterium*-mediated transformation in plants. The prolonged co-cultivation period had a negative effect on the transformation of nodal explants (Table 3). *Agrobacterium* contamination increased with increasing co-cultivation period causing severe damage to the nodal explants. Tissue necrosis of explants resulted subsequently from the overgrowth of bacteria. The problem of excessive overgrowth of *Agrobacterium* with increasing co-cultivation period has been reported earlier by Pandey et al. [17] in *W*. *somnifera*. The frequency of GUS expression was maximum for explants co-cultivated for 3 days (64%) [Fig 3]. The previous report on *W*. *somnifera* transformation employed a co-cultivation period of 5 days for leaf explants-derived from the greenhouse plants [17].

### Effect of acetosyringone on transient GUS expression

Plant specific phenolic compounds that induce the expression of *Agro vir* genes are important for gene transfer [32]. Acetosyringone is suggested as the potent phenolic *vir* gene inducer, containing an unsaturated lateral chain which increases virulence induction and also transformation efficiency [33]. In the present investigation, a low frequency of (64%) nodal explants showed GUS expression when acetosyringone was excluded from the co-cultivation medium (Table 3). The highest frequency (74%) of GUS expression was achieved on WsSIM+AS containing 150 μM AS (Table 3; Fig 3i). The presence of higher concentration of AS (above 175 μM) turned medium yellowish brown and also affected the growth of nodal explants (data not shown). Similar results were obtained in *Eleusine coracana* transformation [34]. Earlier studies reported that lower concentrations of AS (below 100 μM) could not increase bacterial virulence, while higher concentrations of AS (500 μM) was found lethal to bacteria and explants growth [35]. The difference in the requirement of AS for successful transformation of *W*. *somnifera* may be due to the variation in the inoculation and co-cultivation duration and also the competence of target tissue.

### Effect of sonication time on transient GUS expression

In the present study, we have evaluated the effect of sonication time with a constant frequency of 30 KHz on nodal explants of *W*. *somnifera* for the first time to create microwounding to sufficiently produce phenolic compounds for *vir* gene induction. Table 3 and Fig 3k–3o show that the sonication treatment was very effective in increasing transient GUS expression frequency. The frequency of GUS expression increased significantly to a maximum of 100% with increase in sonication treatment from 5 to 25 sec. The explants showed GUS expression (78%) at the regeneration sites when sonication treatment given for 20 sec (Table 3; Fig 3n). At lower sonication treatments (5 and 10 sec), the GUS foci at the cut ends of explants were well defined, corresponding to probably one or a collection of many. A diffuse GUS expression was observed all over the surface of explants, making the quantification of the number of foci difficult with increased sonication treatment for more than 15 sec (Fig 3o). Moreover, when increasing the sonication treatment time, the meristems of explant were damaged. This investigation indicated that longer sonication treatment severally affected the viability of regenerating cells and hence, 10 sec sonication was selected for microwounding procedure. At 10 sec sonication time, a large number of microwounds were produced across the tissue, which permitted the *Agrobacterium* to penetrate deeper and more completely throughout the tissue as compared to the natural infection obtained during co-cultivation, thus improving the bacterial colonization and infection of the tissue [36]. Trick and Finer [37] observed under SEM that ultrasound treatment produced small wounds, which allowed *Agrobacterium* access to internal plant tissue. Sahoo and Jaiwal [38] reported that the sonication treatment has the potential to increase transformation efficiency by improving penetration of *Agrobacterium* cells into the cell layers beneath the epidermis of the cotyledonary node region. SAAT has been playing a vital role in improving the transformation frequency and provided efficient delivery of T-DNA into cells in many plant species [39, 40], especially those that are typically more recalcitrant to *Agrobacterium*-mediated transformation [37].

### Effect of vacuum infiltration on transient GUS expression

**Fig 4:**
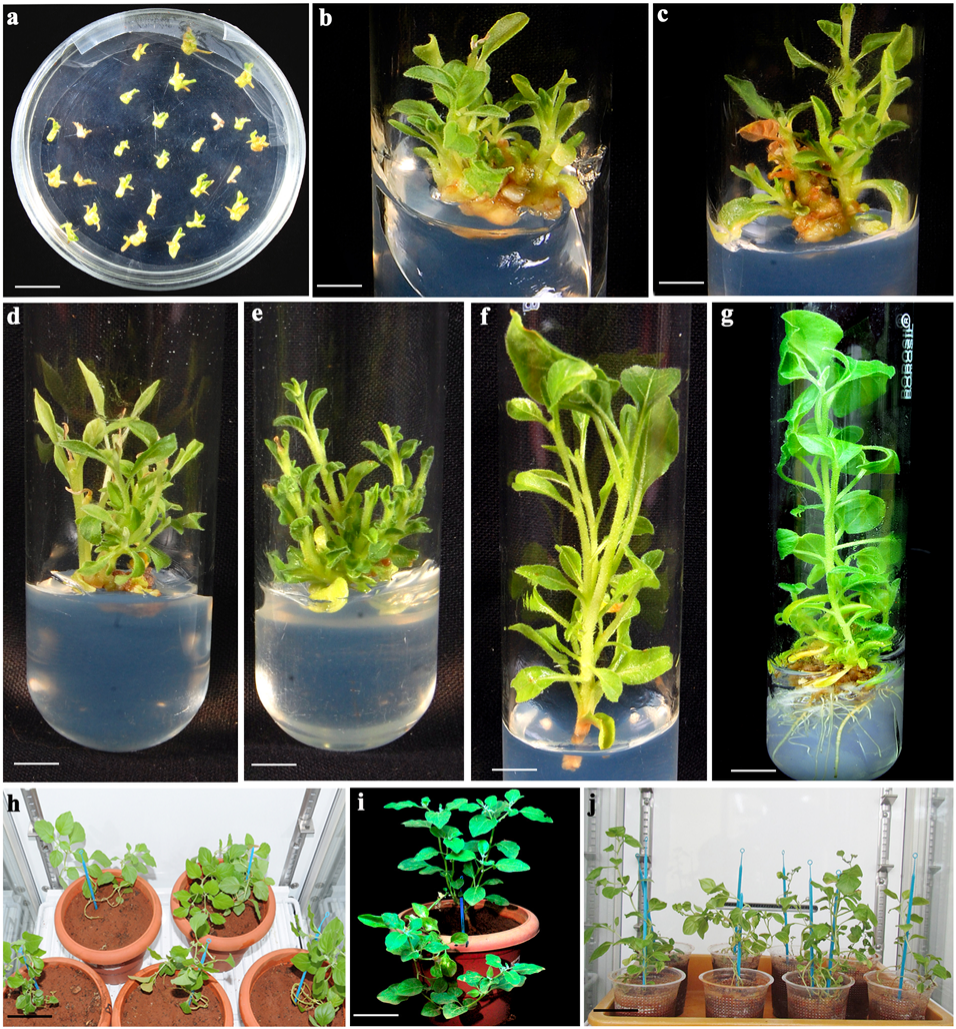
Regeneration of putative transgenic plants of *W*. *somnifera* via *Agrobacterium*-mediated transformation of nodal explants. a. 6 day-old nodal explants pre-cultured on WsSIM; b and c, multiple shoot induction from nodal explants after 15 days of culture on WsSSM-I after inoculation with *A*. *tumefaciens* LBA4404; d and e, multiple shoot proliferation from nodal explants on WsSSM-II after 15 days of culture; f, elongated transgenic shoot cultured on WsSSM-III after 15 days; g, rooting of elongated putative transgenic plantlet on WsRSM; h, T_0_ transgenic plants growing in the controlled environmental growth chamber; I, flowering of T_0_ transgenic plant in the greenhouse; j, T_1_ transgenic plants growing in the controlled environmental growth chamber

Vacuum infiltration treatment was performed in LWsSIM on nodal explants pre-cultured for 6 days. Of different time intervals tested, a 10 min vacuum infiltration resulted in 81% transient GUS expression frequency as recorded on the basis of number of nodal explants showing GUS foci at the regeneration sites (Table 3; Figs 4q and 3p–3s). The number of GUS foci appeared to be quite variable among the nodal explants. The GUS foci were well defined, corresponding to a collection of small individual spots when lower vacuum infiltration treatment of 10 min was applied, beyond which a diffuse GUS expression was observed all over the surface of the explants, making the counting of the number of foci difficult. Increasing the vacuum infiltration treatment time beyond 15 min, the nodal explant’s meristems were injured. Vacuum infiltration has been used to enhance stable transformation of many recalcitrant plants such as *Cicer arietinum* [41], *Vigna unguiculata* [42], and *Leptadenia pyrotechnica* [43].

### Combined effect of sonication and vacuum infiltration on transient GUS expression

A combination of sonication and vacuum treatment was used to enhance transformation efficiency still further. For transformation of *Withania*, the nodal explants were first subjected to a brief period of sonication for 10 sec in LWsSIM followed by vacuum infiltration for 10 min. This kind of treatment, when combined with optimized time duration, exhibited the nodal explants expressing higher frequency (84%) of GUS foci at the regeneration sites (Table 3; Fig 3t). These optimal levels of both the treatments did not affect the nodal explant’s ability to produce multiple shoots. Therefore, we suggest the combined treatments of sonication and vacuum infiltration for increasing transformation efficiency in *W*. *somnifera*. Park et al. [44] used 5 min sonication coupled with 5 min vacuum infiltration in transgenic *Raphanus sativus*. Liu et al. [36] reported that combined effect of sonication and vacuum infiltration improved the transformation efficiency in *Phaseolus vulgaris*. De Oliveira et al. [45] noted that the association of 2 sec sonication with 10 min vacuum infiltration increased the transformation frequency of *Citrus* sps.

### Effect of thiol compounds on transient GUS expression

In the present study, tissue browning was noticed from the bottom region of explants following co-cultivation with *Agrobacterium*. This led to poor regeneration of transformed shoots. An attempt was made to focus on to reduce tissue browning and to improve transformed shoot’s regeneration of explants by adding thiol compounds in co-cultivation medium. Transient GUS expression was recorded in the co-cultured nodal explants on WsSIM+L-Cys medium containing varying concentrations of L-cysteine (Fig 3u–3y). The GUS histochemical expression was recorded only in the apical region of the nodal explants, where the meristematic cells were present at the end of I^st^ selection cycle. Inclusion of L-cysteine at 100 mg/l in the WsSIM medium (WsSIM+L-Cys) improved transient GUS expression frequency (87%) at the apical region of the nodal explants (Table 3; Fig 3u). Morphologically, the appearance of nodal explants co-cultured on WsSIM+L-Cys medium was also improved only up to 100 mg/l L-cysteine; especially less browning was observed on the physically wounded regions on the surface of nodal explants. The co-cultured explants on the same medium containing a higher concentration of L-cysteine (at 200 mg/l and above) led to tissue necrosis and dechlorophyllation followed by bleaching at the end of 1^st^ selection cycle. Frame et al. [46] stated that the increase in stable transformation efficiency observed with 400 mg/l L-cysteine treatment was associated with a decrease in the proportion of embryos giving rise to embryogenic callus compared with control. In the present study, we have observed higher GUS expression frequency (87%) by utilizing L-cysteine concentration as low as 100 mg/l in WsSIM+L-Cys (Table 3; Fig 3u). Olhoft and Somers [47] documented that addition of L-cysteine to co-cultivation medium significantly improved the frequency of GUS expression in cotyledonary node explants of soybean.

Heath [48] reported that when the explants undergo wounding, pathogen infection and environmental stresses throughout co-cultivation with *Agrobacterium*, wound- and pathogen-defense response pathways are active in plant cells. The phytoalexin and secondary metabolites produced by the defense mechanism are serving as repellants or fungicidal/bactericidal agents and by inducing cell death in wounded and infected plant tissue such that a barrier of dead cells is created to protect the adjacent healthy tissue. Hence, in order to overcome these limitations in the present study, thiol compounds were employed in the medium for increasing transformation frequency in *W*. *somnifera*. Inclusion of L-cysteine in the co-cultivation medium improved stable transformation from 0.2% to 5.9% in soybean transformation [49]. Komari and Kubo **[** 50] reported that the main hurdle in *A*. *tumefaciens-*mediated transformation in maize may not be the infection step but may be the recovery of the cells that have integrated the T-DNA into their chromosomes. Cell death caused by hypersensitive response of maize scutellum cells was minimized during the *A*. *tumefaciens* infection when L-cysteine was included in the co-cultivation medium. Frame et al. [46] recorded that the level of higher transformation frequency was achieved by the supplementation of L-cysteine in the co-cultivation medium in maize transformation.

To investigate the effect of STS on the *Agrobacterium* transformation efficiency of nodal explants of *W*. *somnifera*, different concentrations of STS were included in the co-cultivation medium. The addition of STS to the co-cultivation medium consistently resulted in a significantly greater frequency of GUS expression than control. A concentration of 125 mg/l STS, GUS expression frequency was improved to 82% (Table 3; Fig 3c1). Olhoft et al. [20] reported that 248 mg/l STS significantly improved the frequency of transformation in soybean cotyledonary node cells.

Of different concentrations of DTT analyzed, nodal explants treated with 75 mg/l DTT showed better response in transient GUS expression frequency (78.8%) (Table 3; Fig 3h1). Similar to L-cysteine and STS, treated-explants at higher concentrations of DTT exhibited dechlorophyllation at the end of I^st^ selection cycle (data not shown). Olhoft et al. [20] demonstrated that 154 mg/l DTT improved the scoring and percentage of GUS expression in cotyledonary node explants of soybean.

The inclusion of L-cysteine, STS or DTT to the WsSIM during co-cultivation increased T-DNA delivery, GUS expression reduced tissue damage and browning of nodal explants. The addition of 100 mg/l L-cysteine, 125 mg/l STS and 75 mg/l DTT to WsSIM+AS significantly enhanced transient GUS expression frequency (90%; Table 4; Fig 3k1). The association of three optimal concentrations of thiol compounds with optimal levels of PGRs, MES buffer, AS and spermidine greatly improved the transient GUS expression of nodal explants. Olhoft et al. [51] demonstrated that the combined effect of DTT, STS and L-Cys on co-cultivation medium significantly produced GUS positive shoots from cotyledonary node explants of soybean and further stated that combining thiol compounds resulted in a synergistic increase in the regeneration of transformed plant, without non-transformed ‘escapes’ in a relatively shorter period of culture.

**Table 4:** Transformation frequency of shoots regenerated from nodal explants of *W*. *somnifera* in WsSIM+L-Cys+STS+DTT+AS under three selection pressure for recovery of transgenic shoots.

Total No. of infected explants	I selection cycle		II selection cycle		III selection cycle		Transformation frequency (%)
	Explants responding(%)	No. of transgenic shoots	Explants responding (%)	No. of transgenic shoots	Explants responding (%)	No. of transgenic shoots	
50	90.0	-	84	32	78	26	52

Transformation frequency = Total No. of transgenic shoots / Total No. of infected explants ×100

### Selection, regeneration and rooting of transformants

**Fig 5:**
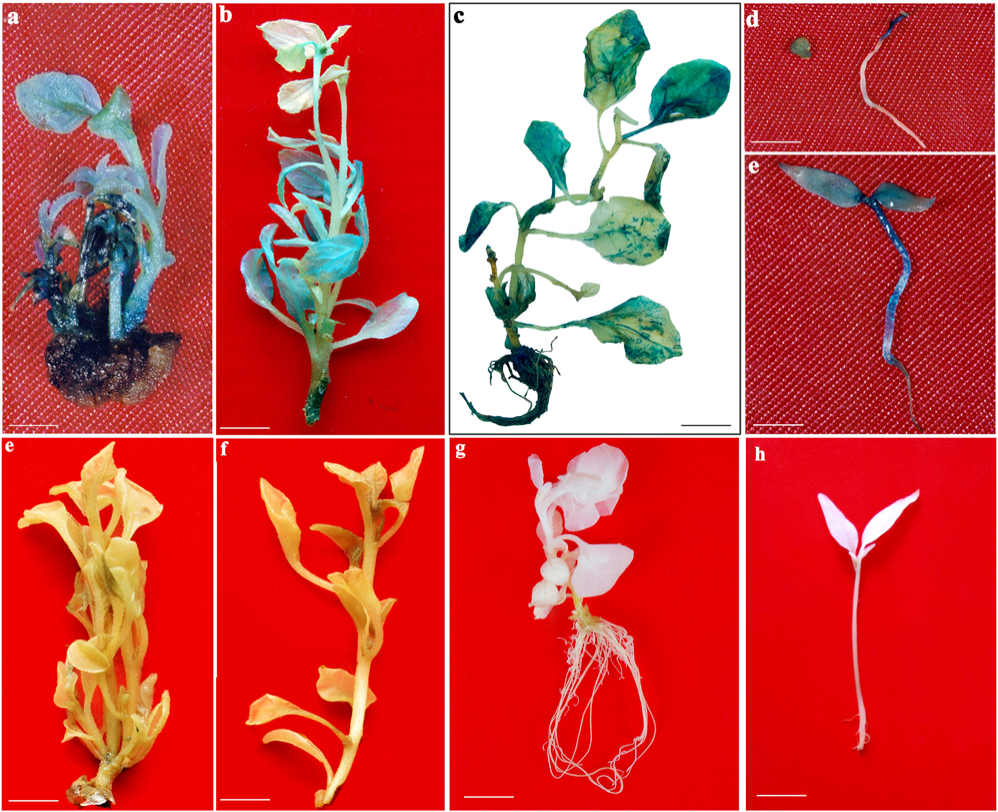
Stable *gusA* gene expressions in regeneration of transgenic plants of *W*. *somnifera* via *Agrobacterium*-mediated transformation using pCAMBIA 2301. a. Transformed cluster of multiple shoots from nodal explants reared on WsSSM-II after 30 days of culture, b. Elongated transgenic shoot cultured on WsSSM-III after 45 days of culture, c. Transformed full plantlet, d and e. Transformed T1 progeny seed and seedlings, f-h. Respective control plants and seedlings.

Optimized transformation procedures (consisting of 6 day pre-culture, 10 sec sonication+10 min vacuum infiltration, 3 days co-cultivation, OD_600_ 0.2, 150 μM AS, L-Cys at 100 mg/l, STS at 125 mg/l, DTT at 75 mg/l) with passing off Ist selection cycle (WsSSM-I) were employed to stably transform *W*. *somnifera* shoots (Table 4; Figs 4 and 5). The nodal explants that survived on 1^st^ selection cycle were transferred to 2^nd^ and 3^rd^ selection cycles with increasing concentrations of kanamycin. 90% of nodal explants exhibiting GUS were recovered in the 2^nd^ selection medium (WsSSM-II) containing 110 mg/l kanamycin. During the 2^nd^ selection cycle, 84% of GUS nodal explants produced 32 transgenic shoots/explant from the regeneration site. After 15 days in the 2^nd^ selection cycle, the shoots with intact explants were transferred to 3^rd^ selection medium (WsSSM-III) supplemented with 115 mg/l kanamycin. At the end of 3^rd^ selection cycle, 78% of GUS nodal explants produced 26 transgenic shoots/explant (Table 4; Figs 4 and 5). In the present study, three selection cycles with increased concentrations of kanamycin resulted in higher and stable GUS expression efficiency. The regeneration response (78%) and the production of transgenic shoots (26 shoots/explant) were comparatively lower than control. During the selection cycles, the transformed shoots proliferated and elongated (4–5 cm) in same selection medium. The elongated shoots (4–5 cm) were transferred to WsRSM containing 75 mg/l kanamycin for root induction. After 10 days of culture, 22 transgenic shoots exhibited root induction at the basal cut end. In order to improve root induction response, 2 mg/l IBA and 20 mg/l putrescine were included on WsRIM for 10 days. In this case, 19 transgenic shoots developed roots (10 roots/elongated transgenic shoot) and lateral roots (data not shown). It was reported that putrescine along with IBA improved healthy root formation frequency in *W*. *somnfera* [10]. After 10 days in WsRIM, kanamycin (50 mg/l) was included in the WsRSM (Table 5). During this selection period, few roots showed necrosis. In this experiment, stable transgenic elongated roots (5 roots/shoot) were produced from 5 transformed elongated shoots at the end of the experiment. The rooted transformed plants (T_0_) which grew on vermiculite, sand and soil were transferred to same substrate composition in pots and were left in greenhouse. The T_1_ progenies showed stable GUS expression in seedlings and seeds whereas the control seeds and seedlings did not show any GUS expression (Fig 5d and 5e).

**Table 5:** Stable transformation frequency of rooted-elongated shoots of *W*. *somnifera* in WsRSM under three selection pressure for recovery of transgenic plants.

Total No. of transgenic shoots inoculated	I selection cycle		II selection cycle		III selection cycle		Transformation frequency (%)
	Total No. of transgenic shoots survived in selection pressure	No. of transgenic roots/shoot	Total No. of transgenic shoots survived in selection pressure	No. of transgenic roots/shoot	Total No. of transgenic shoots survived in selection pressure	No. of transgenic roots/shoot	
26	10	8	8	6	5	5	10

Transformation frequency = Total No. of transgenic shoots rooted under III selection cycle / Total No. of infected explants ×100

### Molecular confirmation

**Fig 6:**
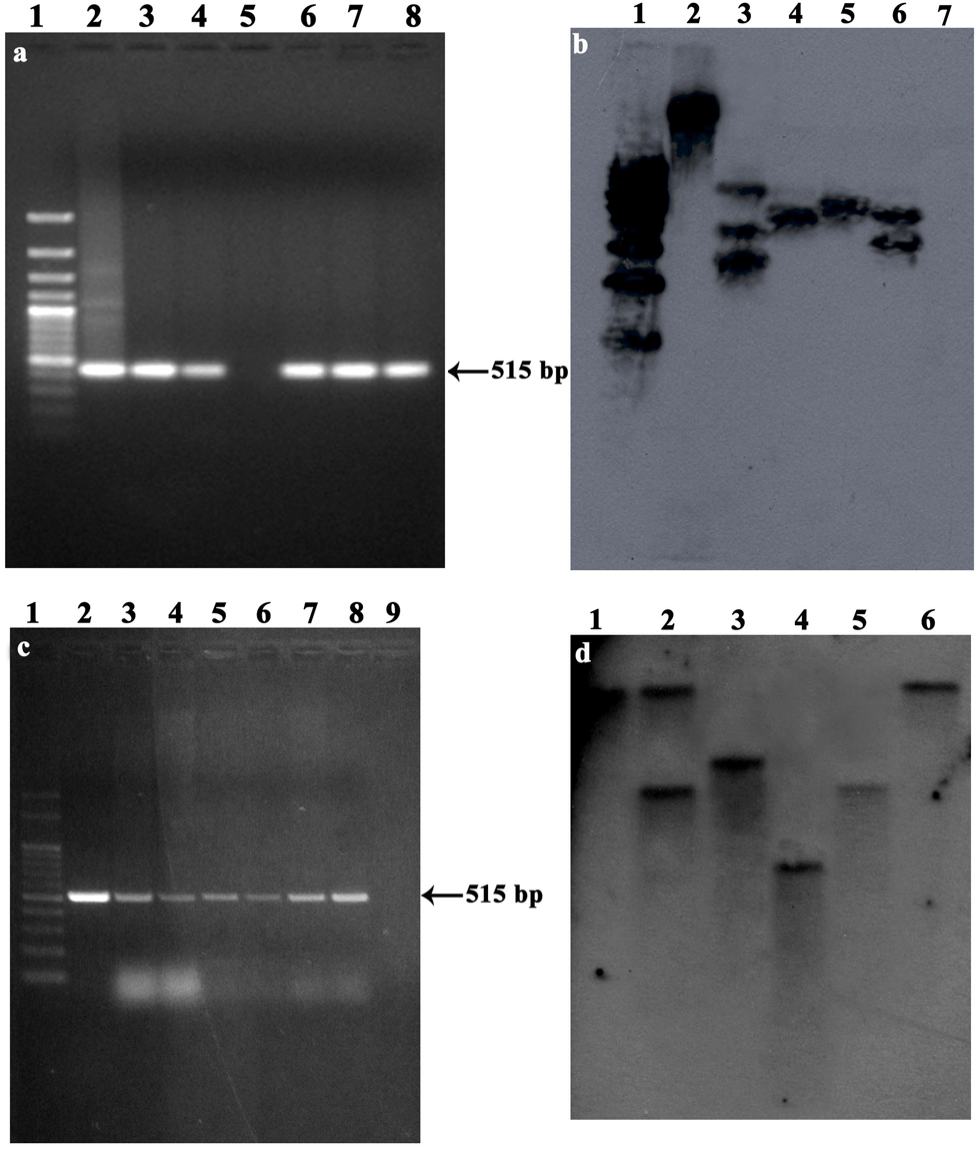
Molecular confirmation of T_0_ and T_1_ transgenic *W*. *somnifera* plants by PCR and Southern blot analysis. a. PCR amplification of the 515 bp fragment of the *gusA* gene in T_0_ transgenic plants. Lane 1–100 bp ladder, lane 2-Plasmid DNA (pCAMIA 2301; positive control), lanes 3, 4, 6, 7, 8- putatively transformed plant DNA, lane 5-non-transformed plant DNA (negative control); b, Southern hybridization analysis to study the T-DNA integration (5391 bp) in T_0_ plants; lane 1 DNA molecular weight marker (λDNA/EcoRI), lane 2-plasmid DNA digested with *Eco*RI (positive control), lanes 3–6 DNA isolated from putative transformed plants, lane 7-non-transformed plant DNA (negative control); c, PCR amplification of the 515 bp fragment of the *gusA* gene in T_1_ transgenic plants. lane 1–100 bp ladder, lane 2-plasmid DNA (pCAMIA 2301; positive control), lanes 3-8- putatively transformed plant DNA, lane 9-non-transformed plant DNA (negative control); d, Southern blot analysis of putatively transformed T_1_ plants. lane 1, pCAMBIA 2301 plasmid (positive control); lanes 2–6, putatively transformed *W*. *somnifera* genomic DNA digested with *Eco*RI and probed with a 515-bp PCR-amplified product of the *gusA* gene.

Kanamycin resistant GUS-positive putative T_0_ plants and their respective controls were subjected to PCR with *gusA* gene specific primers. PCR amplification of T_0_ plants with *gusA* gene specific primers exhibited DNA bands of the expected size of 515 bp (Fig 6a). These amplifications confirmed the presence of transferred expected gene fragment in the transgenic plants. In the case of control, none of the gene fragments was not detected in PCR amplification. The genomic DNA isolated from transformed plants (T_1_), non-transformed plants, and plasmid pCAMBIA 2301 was used as template DNA for PCR amplification of *gusA* gene. The presence of amplified fragment of 515 bp in samples from transformed plants (T_1_) and plasmid confirmed the presence and integration of *gusA* gene (Fig 6c). Amplification of these fragments was not observed in non-transformed control plants.

To confirm *gusA* gene integration and copy number in *W*. *somnifera* genome, Southern blot analysis was performed on total genomic DNA isolated from the leaves of GUS positive shoots (T_0_ and T_1_) and non-transformed plants (Fig 6b–6d). Genomic DNA and pCAMBIA 2301 were digested with EcoRI, which recognizes a single site within the T-DNA. Because the T-DNA region of pCAMBIA 2301 has only one EcoRI site, digestion of genomic DNA with EcoRI and probing with the 515-bp PCR-amplified product of *gusA* generates a unique fragment for each integrated copy. Therefore, the number and position of the bands are expected to reflect the integration of *gusA* into different positions and provides an estimate of the copy number in the genome of transformed T_0_ and T_1_ plants. The position of each band differed from plantlet to plantlet, thus indicating independent transformation events and random integration. The transformed *W*. *somnifera* plants showed either a single copy or two copies of *gusA* integrated into the genome (Fig 6b–6d).

### Quantification of withanolides

**Fig 7:**
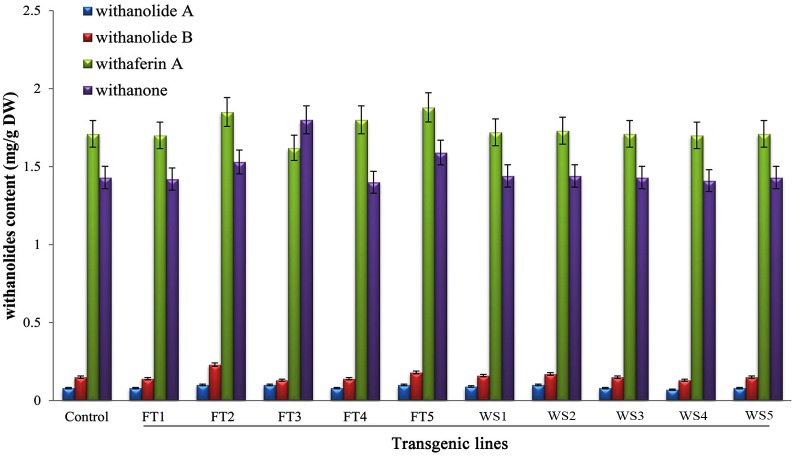
Quantities of withanolide A, withanolide B, withaferin A and withanone contents in T_0_ (FT1-FT5) and T_1_ (T1–T5) transgenic lines of *W*. *somnifera* and its control plant. Data represents mean ± standard error of three replicates.

The quantities of major withanolides found in the transgenic lines [T_0_ (5 plants-FT1, FT2, FT3, FT4 and FT5) and T_1_ (5 plants-WS1, WS2, WS3, WS4 and WS5)] and non-transformed plants are presented in Fig 7. The withanolides contents were varied depending upon the transgenic lines (T_1_ or T_0_ plants). The non-transgenic plant exhibited 0.08 mg/g DW withanolide A, 0.15 mg/g DW withanolide B, 1.71 mg/g DW withaferin A and 1.43 mg/g DW withanone. In T_0_ plants, maximum content of withanolide A (0.1 mg/g DW) was recorded in FT2, FT3 and FT5 lines; withanolide B (0.23 mg/g DW) in FT2 line; withaferin A (1.88 mg/g DW) in FT5 line and withanone (1.8 mg/g DW) in FT3 line (Fig 7). These levels were considerably higher (withanolide A 1.25-fold, withanolide B 1.53-fold, withaferin A 1.09-fold and withanone1.25-fold higher respectively) when compared to non-transgenic plants. In T_1_ plants, higher contents of withanolides were recorded (withanolide A (0.1 mg/g DW in T2 line), withanolide B (0.17 mg/g DW in T2 line), withaferin A (1.73 mg/g DW in T2 line) and withanone (1.44 mg/g DW) in T1 and T2 line than control [Fig 7]. Pathogen infection in plants led to changes in secondary metabolism based on the induction of defence programmes [52]. *Agrobacterium* infection in *Brassica rapa* suppressed the production of secondary metabolites such as flavonoids and phenylpropanoids when compared to intact plants [52]. Whereas, in the present study, withanolides profile were recorded slightly higher. From this observation, the capability of transgenic plants on withanolides biosynthesis was related to *Agrobacterium* infection.

## Conclusion

The present study demonstrated the production of transformed plants of *W*. *somnifera* via *Agrobacterium*-mediated transformation. A transformation frequency of 10% was obtained on the basis of detection of GUS staining of kanamycin resistant putative transformed plants. Out of several factors that were examined, bacterial density, physical wounding method and addition anti-necrotic compounds in the medium were the three major factors that promotsed an efficient transformation system for *W*. *somnifera*. The meristem based transformation system developed in the present study may be used for metabolic engineering of *farnesyl pyrophosphate synthase*, *squalene synthase*, *squalene epoxidase* and *sterol methyltransferase* genes into the genome of *W*. *somnifera*.
